# Differences in the spatial fidelity of evoked and spontaneous signals in the degenerating retina

**DOI:** 10.3389/fncel.2022.1040090

**Published:** 2022-11-07

**Authors:** Maya Carleton, Nicholas W. Oesch

**Affiliations:** ^1^Department of Psychology, University of California, San Diego, La Jolla, CA, United States; ^2^Department of Ophthalmology, University of California, San Diego, La Jolla, CA, United States; ^3^The Neurosciences Graduate Program, University of California, San Diego, La Jolla, CA, United States

**Keywords:** retina, blindness, retinal prosthetics, retinitis pigmentosa, vision restoration, electrical stimulation, retinal ganglion cell

## Abstract

Vision restoration strategies aim to reestablish vision by replacing the function of lost photoreceptors with optoelectronic hardware or through gene therapy. One complication to these approaches is that retinal circuitry undergoes remodeling after photoreceptor loss. Circuit remodeling following perturbation is ubiquitous in the nervous system and understanding these changes is crucial for treating neurodegeneration. Spontaneous oscillations that arise during retinal degeneration have been well-studied, however, other changes in the spatiotemporal processing of evoked and spontaneous activity have received less attention. Here we use subretinal electrical stimulation to measure the spatial and temporal spread of both spontaneous and evoked activity during retinal degeneration. We found that electrical stimulation synchronizes spontaneous oscillatory activity, over space and through time, thus leading to increased correlations in ganglion cell activity. Intriguingly, we found that spatial selectivity was maintained in *rd10* retina for evoked responses, with spatial receptive fields comparable to *wt* retina. These findings indicate that different biophysical mechanisms are involved in mediating feed forward excitation, and the lateral spread of spontaneous activity in the *rd10* retina, lending support toward the possibility of high-resolution vision restoration.

## Introduction

Diseases such as retinitis pigmentosa and age-related macular degeneration are among the most common forms of degenerative blindness (The Eye Diseases Prevalence Research Group, 2004). These diseases also serve as important model systems for neurodegeneration owing to the retina’s experimental tractability (Tirassa et al., 2018; Trapani and Auricchio, 2018). Recent advances in optoelectronics have enabled high-resolution retinal prosthetics (Ha et al., 2016; Yang et al., 2016; Bosse et al., 2018; Edwards et al., 2018; Palanker et al., 2020), new gene therapy strategies also show promise (Trapani and Auricchio, 2018; Wood et al., 2019; Sahel et al., 2021; Cehajic-Kapetanovic et al., 2022), and advances in stem cell research has allowed for the transplantation of new photoreceptors (Ludwig and Gamm, 2021; Ribeiro et al., 2021; Zerti et al., 2021); however, there is much concern about how disease related changes impact the fidelity of restored vision (Dräger and Hubel, 1978; Sauvé et al., 2001; Stasheff, 2008, 2018; Toychiev et al., 2013; Yee et al., 2014; Ivanova et al., 2016; Jones et al., 2016; Telias et al., 2019, 2022; Zerti et al., 2021). Although, some interactions between disease related changes and prosthetic vision restoration have been studied in the past (Ryu et al., 2011; Yee et al., 2012; Goo et al., 2015; Cho et al., 2016; Stutzki et al., 2016; Ahn et al., 2022), many important questions remain unresolved, such as how degeneration changes spatiotemporal processing in retinal circuitry.

Aberrant spontaneous activity has long been reported in many animal models of retinitis pigmentosa (Ye and Goo, 2007; Margolis et al., 2008; Stasheff, 2008). Spontaneous activity typically manifests as rhythmic bursting or oscillatory activity. Previous studies, most commonly in the *rd1* or *rd10* (Chang et al., 2002) mouse models, have shown significant variability in the characteristics of spontaneous activity, such as the dominant frequency, as well as the underlying changes that could mediate this aberrant activity (Margolis and Detwiler, 2011; Biswas et al., 2014; Gehlen et al., 2020). Putative mechanisms for spontaneous activity can be broadly divided into inner and outer retina mechanisms, which are not mutually exclusive [for review see, Euler and Schubert (2015) and Trenholm and Awatramani (2015)]. In the outer retina, remnant cones, rod bipolar cells, and horizontal cells show membrane oscillations at a rhythm between 1 and 3 Hz, mediated by gap junctions and GABAergic synapses (Haq et al., 2014). In the inner retina, the AII amacrine/ON-cone bipolar cell (AII-CBC) gap junction network has characteristic oscillations around 10 Hz in *rd1* and 2–8 Hz in *rd10* mice (Trenholm et al., 2012). Both inner and outer circuits utilize inhibitory interneuron/gap junctional networks to mediate spontaneous activity (Euler and Schubert, 2015). Other work has suggested a third mechanism postsynaptic to the ganglion cells (Telias et al., 2019), based on the observation that blocking excitatory and inhibitory inputs is not always sufficient to block all spontaneous activity (Borowska et al., 2011; Margolis and Detwiler, 2011; Sekirnjak et al., 2011; Haq et al., 2014; Barrett et al., 2016). Regardless of the specific locus of the oscillatory network, ongoing spontaneous activity will clearly degrade the signal to noise of retinal prosthetic evoked stimulation (Ivanova et al., 2016; Yoon et al., 2020). Moreover, the retina dedicates significant resources to decorrelate signals through both center-surround receptive field properties and other mechanisms (Pitkow and Meister, 2012; Franke et al., 2017), consistent with the efficient coding hypothesis (Barlow, 1961). While this has been well-characterized in wild-type (*wt*) retina, little is known about the effects of degeneration on efficient population encoding, specifically in response to electric stimuli.

Although, much work has focused on the implications for spontaneous activity, fewer studies have examined how evoked responses are altered during degeneration, and results vary widely (Lorach et al., 2015a; Stutzki et al., 2016; Jalligampala et al., 2017; Ho et al., 2020; Sekhar et al., 2020; Ahn et al., 2022). In addition, few have investigated how evoked activity alters the properties of spontaneous activity. To examine this, we compared spontaneous activity, alone and following electrical stimulation, in both *wt* and *rd10* retina. Our data shows that electrical stimulation can synchronize spontaneous oscillatory activity in retinal ganglion cells (RGCs) through time and over retinal space in the *rd10* retina, but not *wt*. This effect was worsened when inhibition was blocked. Intriguingly, the synchronization of RGC spontaneous activity over greater distance in *rd10* retina cannot be explained by a general increase in *rd10* receptive fields, as electrical receptive fields were narrower in *rd10* retina than *wt*.

## Materials and methods

### Retina explant and loose patch electrical recording

All experimental methods and animal care procedures were conducted in accordance with NIH guidelines and were approved by the University of California, San Diego Institutional Animal Care and Use Committee. In total we collected data from 141 RGCs from 18 *rd10* mice and 104 RGCs from 16 C57BL/6J mice, of either sex (P60-180). Animals were anesthetized with isoflurane, euthanized by cervical dislocation, enucleated, and their retinas were dissected free and maintained in Ames medium oxygenated and equilibrated with 95% O_2_, 5% CO_2_. Retina pieces, approximately 2 mm × 2 mm, placed over stimulating electrodes on the bottom of the custom recording chamber, ganglion cell side up. The chamber was placed under an upright microscope and perfused with Ames solution (4 ml/min) at 35°C. RGCs were visualized and targeted using IR differential interference contrast video microscopy. Contact between the outer portion of the retina and the electrode array surface was also confirmed with microscopy. Stimulation was delivered on the nearest electrode to the target RGCs often within ∼30 μm in the x-y plane.

**FIGURE 1:**
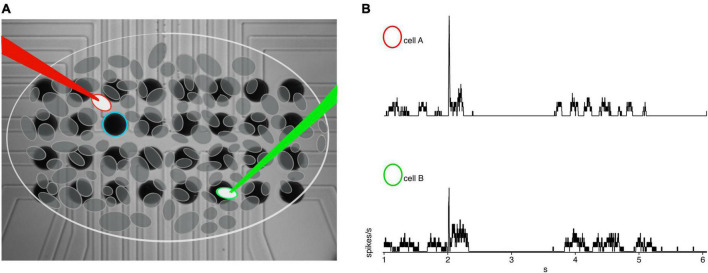
Example recording set up. **(A)** 4 × 8 30 μm iridium oxide electrode grid with schematic representation of RGC’s overlaid. The white circle with red outline indicates the cell of interested closest to the blue stimulating electrode, with a red loose patch recording electrode. The green circle is the secondary cell of interest recorded *via* the green loose patch electrode. **(B)** Example rates from cell A (red circle) and cell B (green circle) in rd10 retina.

Sputtered iridium oxide film (SIROF) stimulating electrode arrays were fabricated, as described previously, on a borosilicate glass disk, which formed the bottom of the recording chamber (Damle et al., 2021). Briefly, the array consisted of a 4 × 8 rectangular grid of 30 μm diameter SIROF electrodes spaced 50 μm apart from center to center (Figure 1A). SIROF electrodes were formed by reactive DC sputtering to a thickness of 600 nm over indium tin oxide (ITO) traces. ITO traces terminated in gold contact pads at the edge of the disk for connection to a 32-channel RHS2000 stim and recording system (Intan Technologies, Los Angeles, CA, USA). ITO traces were insulated with a 200 nm layer of SiNx. Charge storage capacity was typically 12 and 17 mC/cm^2^, for anodal and cathodal charge, respectively. Charge injection limits were determined to be near 3 mC/cm^2^, thus for a 30 μm diameter electrode we used an upper limit of 20 nC, per phase. Charge-balanced, anodic first, square, biphasic current pulses were generated on the RHS2000, triggered by our acquisition software, and delivered to an individual stimulating electrode nearest to the cell of interest. Individual stimulus pulses were delivered every 10 s and repeated five times per condition.

Recording electrodes were pulled from borosilicate capillary glass to have a final resistance of 4–5 MW and filled with Ames medium. Loose-patch recordings were made from ganglion cells and action potentials were recorded in voltage-clamp mode using a Multiclamp 700b (Molecular Devices) patch-clamp amplifier. Signals were filtered at 4 kHz (4-pole Bessel), digitized at 20 kHz with an ITC-18 (HEKA Electronik) data acquisition board and saved to a PC for offline analysis using custom acquisition software in IgorPro8 (WaveMetrics, Lake Oswego, OR, USA).

Distances were measured using Scientifica LinLab2 and IR-DIC microscopy. The primary cell was positioned in the center of the field-of-view and marked as the coordinate zero point. The stage was then moved to the center of the secondary cell and the x,y coordinate from LinLab2 was recorded. The x, y coordinates were translated into Euclidian distance for analysis. For paired recording experiments, the primary cell was within 35 μm in the x-y plane of the stimulating electrode, given the stimulating electrode spacing, while the secondary cell was often farther depending on the specific geometric relationship. Inter-pair distances were measured using the distance between the center of the cell somas (Figure 1A).

Pharmacological agents were added to the superfusate. Inhibition was blocked with a cocktail of 5 μM strychnine (Sigma-Aldrich, Saint Louis, MO, USA), 5 μM SR-95531, and 50 M and TPMPA (Tocris BioScience, Bristol, UK), to block glycine, GABA_*A*_, and GABA_*C*_ receptors, respectively. Gap junctions were blocked using 100 μM Meclofenemic acid (MFA, Sigma-Aldrich, Saint Louis, MO, USA) in combination with inhibitory antagonists. Excitation was blocked with 10 μM NBQX, and 50 μM AP5 (Tocris BioScience, Bristol, UK) to block AMPA/kainite and NMDA receptors, respectively.

### Analysis

Data processing and statistical analyses were performed in IgorPro 8. Spikes were identified by thresholding the mean-subtracted, first derivative of the raw data, with a threshold that was > 5x the peak-to-peak noise amplitude. Spike rates were then computed using a 10 ms boxcar average (Figure 1B). Individual cell responses were calculated from the average of the five repeats. Cross correlations were performed in Igor Pro 8 from averaged spike rates for each pair of cells and mean subtracted to obtain the non-normalized cross correlation to exemplify the unique power structure and periodicity of paired cells for all experimental conditions. Receptive field widths were measured as the 1-standard deviation width of the Gaussian fit.

Mutual information analysis was computed in MATLAB (The MATLAB Inc, Natick, MA, USA) using available code from Neuroscience Information Theory Toolbox (Timme and Lapish, 2018). Mutual Information was computed using a matrix of 5 rate sweeps for each cell.


$$\mathrm{MI}\,\mathrm{equation}\,I\,\left(X;Y\right)=\sum_{x\in X,y\in Y}p\,\left(x,y\right)\,\log_{2}\left(\frac{p\,\left(x,y\right)}{p\,\left(x\right)\,p\,\left(y\right)}\right)$$

Continuous data was binned using 2000-point wide uniform bins. Data was classified into four states of spiking levels for each time bin and compared against their simultaneously recorded cell partner with no time lag. Mutual information was outputted in bits for each time bin, with the output matrix being equal in length to the original input data.

## Results

### Correlated activity between retinal ganglion cells

Many but not all RGCs in *rd10* retina have rhythmic spontaneous activity, and there is heterogeneity among spontaneously active RGCs (Margolis et al., 2008; Stasheff, 2008; Menzler and Zeck, 2011; Biswas et al., 2014; Ahn et al., 2022). How spontaneous activity in individual RGCs is related to other nearby RGCs is less clear. Some reports in the *rd10* retina have observed anticorrelated oscillations between on and off ganglion cells (Margolis et al., 2014); however, other reports did not detect any specific phase relationship (Margolis et al., 2008; Stasheff, 2008; Menzler and Zeck, 2011; Biswas et al., 2014; Ahn et al., 2022). Moreover, details of how electrical stimulation modulates the spatio-temporal correlations between spontaneous RGC activity is less well-understood (Goo et al., 2015; Stutzki et al., 2016; Haselier et al., 2017; Ahn et al., 2022).

**FIGURE 2:**
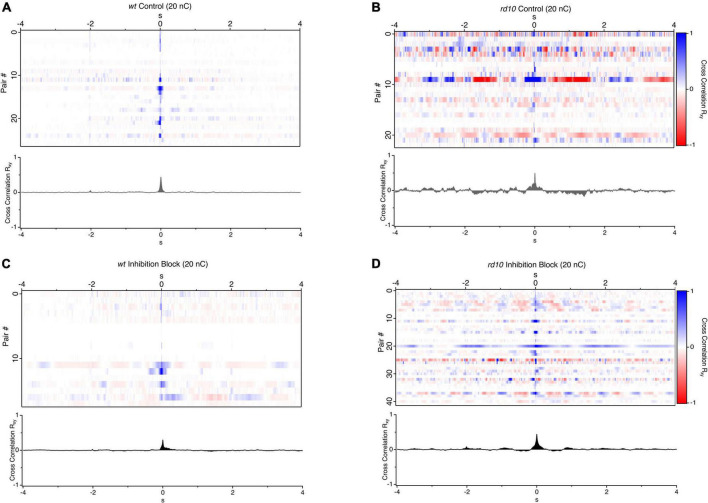
Cross correlations of evoked and spontaneous activity between RGC pairs. **(A)** Heatmaps of spike rate cross-correlations between pairs of RGCs where each line is a pairwise cross-correlation for *wt* (*n* = 27), and **(B)** *rd10* RGCs (*n* = 23) in control conditions and **(C,D)** during application of inhibitory antagonists (5 μM SR-95531, 50 μM TPMPA, and 5 μM strychnine), *wt* (*n* = 18) and *rd10* (*n* = 41). A 20 nC, 1 ms biphasic stimulus was used. Traces below each heatmap are the average cross-correlation for all RGC pairs in each condition.

To examine these questions, we made simultaneous loose-patch recordings from pairs of RGCs in *wt* and *rd10* retina and computed the mean subtracted cross correlations in spiking activity following electrical stimulation consisting of a 20 nC pulse on the nearest electrode, which was typically within 30 μm to the nearest cell (Figures 1A,B). *Wt* RGC pairs showed no consistent correlation outside of the stimulus evoked response which was limited to a peak width corresponding to an evoked response lasting ∼50 ms (Figure 2A). In contrast, cross-correlations between *rd10* pairs showed regular peaks and troughs at times well outside the stimulation-evoked response reflecting periodic spontaneous activity. Although, there was heterogeneity in the patterns of cross-correlograms for individual pairs, when all pairs were averaged together consistent peaks and troughs could still be seen in the average indicating that spontaneous periodic activity could be synchronized by the stimulus pulse (Figure 2B).

Past work has largely converged on a model where a gap junction coupled excitatory network, gives rise to oscillatory spontaneous activity in the *rd10* retina (Euler and Schubert, 2015; Trenholm and Awatramani, 2015, for review) although, studies indicated that inhibition plays a role in modulating this spontaneous activity (Margolis and Detwiler, 2011; Biswas et al., 2014; Gehlen et al., 2020). To examine how inhibition modulates or synchronizes spontaneous activity in response to stimulation, we repeated these experiments during inhibitory neurotransmitter block. For *wt* pairs, inhibition block led to a small increase in the decay of the evoked response and a broadening of the correlation peak at time zero. As expected, there was also an increase in spontaneous firing, which led to an increase in total power in the cross-correlations. However, there was still no consistent pattern of correlation in the activity outside of the stimulus evoked burst. This indicates that elevated spontaneous firing during inhibition block in *wt* pairs is unstructured and independent (Figure 2C). In contrast, the *rd10* pair cross-correlations in the presence of inhibitory antagonists had regular peaks and troughs as reflected in the average cross correlations (Figure 2D) beyond the stimulation evoked burst of activity following stimulation. For both control and inhibition block in *rd10*, peaks and troughs were symmetrically centered on the zero-point indicating that rhythmic activity was in phase between pairs of cells. This phase alignment could be caused by stimulation, or it could result if spontaneous activity was naturally synchronized due to the network properties of the *rd10* retina.

**FIGURE 3:**
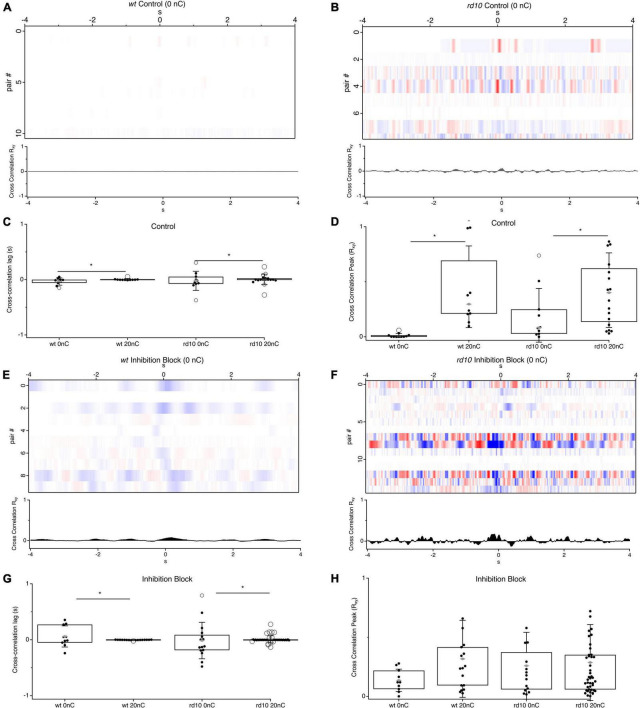
Comparison of cross-correlation between RGC pairs for stimulation and no stimulation conditions. **(A)** Heatmaps of cross-correlations between RGC pairs for *wt* control conditions (*n* = 11) and **(B)** *rd10* (*n* = 10) during no stimulation. **(C)** Box plots for comparison of times of cross-correlation peaks for *wt* and *rd10* RGC pairs at 20 nC and 0 nC. **(D)** Box plots for cross-correlation peak magnitude. **(E,F)** Heatmaps of cross-correlations between RGC pairs for *wt* (*n* = 10) and *rd10* (*n* = 15) during inhibition block (5-μM SR 95531, 50 μM TPMPA, and 5 μM strychnine). **(G,H)** Same as panels **(C,D)**, but during inhibition block. For box plots, filled black circles represent data points, open circles represent outliers, whiskers show 1 standard deviation, gray diamonds represent the mean. *Denotes *p* < 0.05. Traces below each heatmap are the average cross-correlation for all RGC pairs in each condition.

We hypothesized that stronger correlations between *rd10* RGCs was a result of electrical stimulation, given that past work has suggested that the phase and frequency of spontaneous activity is variable between cells (Margolis et al., 2008; Stasheff, 2008; Menzler and Zeck, 2011; Biswas et al., 2014; Ahn et al., 2022). To determine if stimulation was synchronizing spontaneous rhythmic activity between cells, we measured the magnitude of the cross-correlation peak, which should be larger for more synchronous activity, and the time of the peak in the cross-correlation relative to time zero, which should be closer to zero for more synchronous activity. We compared these measures in the absence of electrical stimulation to the cross-correlations measured above during 20 nC stimulation (Figure 3). For *rd10* pairs the variance of the peak time was significantly greater than during stimulation (Figure 3C; Bartletts Variance test, *p* < 0.05). In the absence of stimulation, the peak times were symmetrically distributed around the zero-time point, with a 1 standard deviation width of 172 ms and a mean of 20 ms, compared to a width of 90 ms and mean of 3 ms during stimulation conditions. The decrease in cross-correlation peak time and decrease in temporal spread of peak times demonstrates that stimulation synchronizes rhythmic spontaneous activity. This effect was more pronounced in inhibition block (Figures 3G,H). The variance of the peak time for stimulation decreased further during inhibition block with a standard deviation width of 60 ms and a mean of 8 ms (Figure 3G). The general effect was also seen for *wt* pairs, however, given the low rates of spontaneous activity, the overall magnitude of power in the cross-correlation is near zero (Figure 3A). Even during inhibition block when *wt* RGCs have higher spontaneous firing rates, the magnitude of the cross correlations was still less than *rd10* (Figure 3H, *p* = 0.04, ANOVA). As expected, evoked responses do contribute to the power of the cross-correlation, as shown by the decrease in peak power in the absence of stimulation, however, there is still more power in the *rd10* cross-correlation in the absence of stimulation than for *wt* in both control conditions and inhibition block, indicating that spontaneous activity can drive significant correlations even in the absence of stimulation in *rd10* retina (Figure 3, *p* = 0.041, ANOVA, *p* = 0.021, ANOVA). Together, this demonstrates that changes in the *rd10* circuit drive rhythmic and correlated activity between RGCs, and importantly, that electrical stimulation synchronizes activity between cells with otherwise heterogeneous phase relationships.

### Mutual information as a measure of redundant information

**FIGURE 4:**
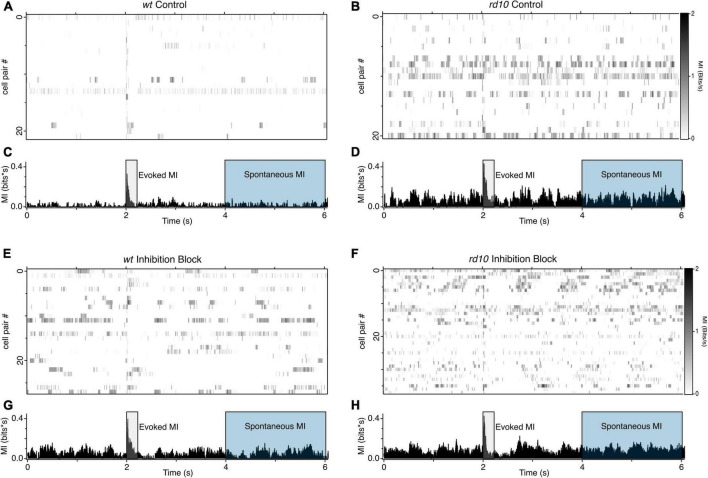
Patterns of MI between RGC pairs **(A)** Heatmaps of MI between RGC pairs for *wt* (*n* = 22) and **(B)** *rd10* (*n* = 21). **(C,D)** Histograms showing the averaged binned MI for all RHC pairs shown in the heatmap above. **(E–H)** Same as **(A–D)**, but during inhibition block (5 μM SR-95531, 50 μM TPMPA, and 5 μM strychnine). *wt* (*n* = 28), *rd10* (*n* = 38). A 20 nC, 1 ms per phase, biphasic stimulation was delivered at time 2 s. Shaded regions show time periods used to integrate evoked and spontaneous MI.

A major implication of increased correlated activity between ganglion cells is that it increases the redundancy of information, thus making visual coding less efficient in the disease state [Puchalla et al., 2005; but see Ruda et al. (2020)]. While correlations are commonly used as a proxy for coding efficiency, weaker correlations do not necessarily imply weaker dependence, particularly for spike trains with a non-Gaussian distribution (Pitkow and Meister, 2012) as is true for most neurons. Moreover, how dependencies evolve after electrical stimulation is lost in the cross correlation and correlation index measurements. To circumvent these limitations, we computed the mutual information (MI) contained in action potential rates between pairs of RGCs. MI quantifies the degree to which the response of one cell predicts the activity of the other, expressed in bits of shared information (Timme and Lapish, 2018). We computed the average MI in 20 ms bins for each pair of cells over five repeats (Figures 4A–D). We compared the MI in periods immediately after the stimulus (2–2.2 s) to measure evoked activity, referred to from here-on as evoked MI, and at later period (2–4 s) post-stimulus to measure the effect of stimulation on spontaneous activity, referred to as spontaneous MI. We chose the 2–4-s post stimulus window as our spontaneous activity MI measure to avoid potential confounds with long-lasting evoked activity with spontaneous activity. We normalized the total integrated MI to the duration of the measurement window.

**FIGURE 5:**
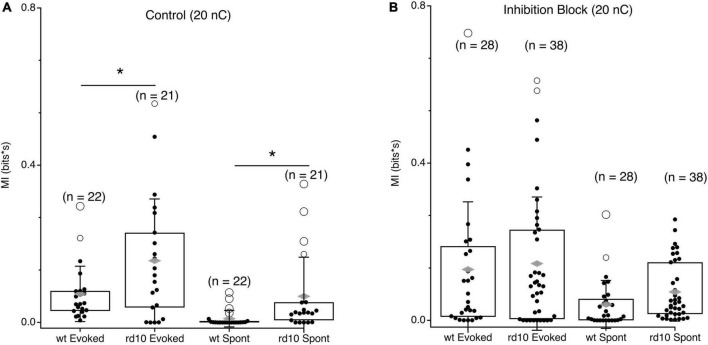
Comparison of evoked and spontaneous MI between *wt* and *rd10* RGC pairs. **(A)** Box plots showing integrated MI between RGC pairs in *wt* and *rd10* retina for evoked and spontaneous time periods. **(B)** Same as panel **(A)**, but in inhibition block (5 μM SR-95531, 50 μM TPMPA, and 5 μM strychnine). Filled black circles represent data points, open circles represent outliers, whiskers show 1 standard deviation, gray diamonds represent the mean. *Denotes *p* < 0.05.

As expected, when the stimulus evokes activity in both cells, MI is high in both *wt* and *rd10* mice during the stimulus evoked time period, however, *rd10* RGCs have higher levels of MI than *wt* (Figure 5A, *p* = 0.024, *T*-test), indicating that redundancy is higher in rd10 retina even for evoked responses. Importantly, MI is significantly higher at times long after the stimulus (Figure 5A, *p* = 0.018, *T*-test), indicating that spontaneous activity in *rd10* RGCs does indeed increase redundancy in the population code.

Given the role of inhibition in decorrelating responses in the retina, we also measured MI during inhibition block. Consistent with inhibition functioning to decorrelate RGC responses during normal function, MI increased between RGC pairs for both evoked and spontaneous spiking in the *wt* retina (Figure 5B). Intriguingly, MI did not significantly increase for *rd10* RGC pairs when inhibition was blocked, for either evoked or spontaneous activity. This could point to a deficit in inhibitory function in the *rd10* retina, or it could indicate that the severity of dysfunction in the excitatory network is too great for inhibition to overcome, although our data cannot resolve these possibilities.

**FIGURE 6:**
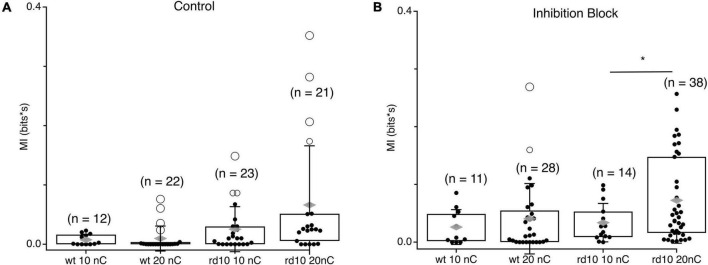
Comparison of spontaneous MI between RGC pairs at different stimulation levels. **(A)** Box plots showing integrated MI between RGC pairs in *wt* and *rd10* retina for spontaneous activity following 10 or 10 nC stimulation. **(B)** Same as panel **(A)**, but in inhibition block (5 μM SR-95531, 50 μM TPMPA, and 5 μM strychnine). Filled black circles represent data points, open circles represent outliers, whiskers show 1 standard deviation, gray diamonds represent the mean. *Denotes *p* < 0.05.

Given our observation that stimulation synchronizes spontaneous activity beyond the evoked response, we hypothesized that the strength of spontaneous MI between RGC could be modulated by stimulation strength, being greater for stronger stimulation (Figure 6). In control *wt* RGC pairs, spontaneous MI was very low given the low rate of spontaneous activity in *wt* and was only 0.002 bits greater for 20 nC stimulation compared to 10 nC stimulation. For *rd10*, spontaneous activity MI was more than twice as large for 20 nC stimulation vs. 10 nC stimulation. Variability in MI was also greater for 20 nC than 10 nC, where some RGC pair MI was modulated by stimulation strength, but other pairs were insensitive. In inhibition block, spontaneous MI generally went up with, consistent with Figure 4, but the percent increase for both *wt* and *rd10* RGC pairs remained consistent (Figure 6B, *p* = 0.013, *T*-test). Despite the heterogeneity in spontaneous MI between *rd10* RGC pairs, we conclude that stimulation can modulate the amount of coordinated activity between many RGCs in the *rd10* retina.

These results clearly demonstrate that the fundamental properties of population coding are disrupted in *rd10* retina, beyond the addition of correlated spontaneous activity. This also provides further support for the use of MI to evaluate coordinated activity between RGCs compared to cross-correlations. Although measures of peak cross-correlation power increased modestly in the presence of inhibition blockers for *wt*, analysis using MI reveals a more pronounced role of inhibition than were revealed by cross-correlations alone.

It is widely accepted that gap junctions are required to support spontaneous activity during degeneration, although whether gap junctions coupling is abnormal during degeneration is location specific (Euler and Schubert, 2015). Briefly, aberrant gap junction coupling maybe present in the outer retina, but gap junction coupling in the inner AII Amacrine On-Cone Bipolar cell (AII-CBC) network is likely unchanged (Borowska et al., 2011; Trenholm et al., 2012; Menzler et al., 2014). A few reports also support abnormal gap junction coupling between RGCs as a locus for spontaneous activity (Telias et al., 2019, 2022; Denlinger et al., 2020).

**FIGURE 7:**
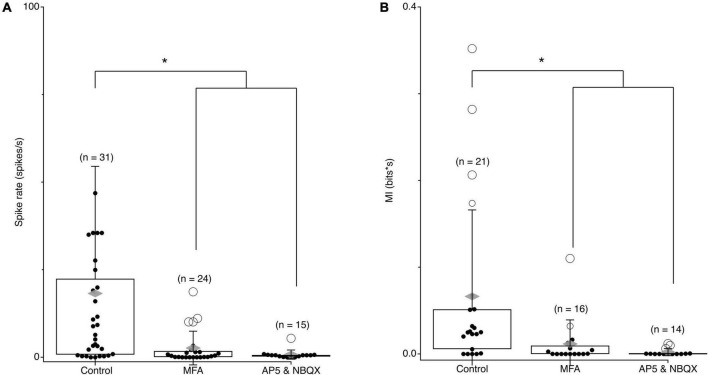
Spontaneous MI between RGC pairs is blocked by gap junctions and excitatory synapses. **(A)** Average spontaneous firing rates for *rd10* RGCs pairs in control conditions, 100 μM MFA, and 50 μm AP5 and 10 μm NBQX. **(B)** Average spontaneous MI between *rd10* RGC pairs in control conditions, 100 μM MFA, and 50 μM AP5 and 100 μM NBQX. Filled black circles represent data points, open circles represent outliers, whiskers show 1 standard deviation, gray diamonds represent the mean. *Denotes *p* < 0.05.

To examine what changes may be involved in driving correlations in spontaneous activity, we first confirmed that spontaneous activity and synchronized activity are eliminated when gap junctions are blocked. Spontaneous spike rates in *rd10* RGCs were significantly reduced in the presences of 100 μm MFA (Figure 7A; *p* = 6.17e-08), while they remained unchanged in *wt* retina (data not shown). Spontaneous MI between RGC pairs was also decreased in the presences of MFA (Figure 7B; *p* = 0.0034). To check if spontaneous activity arose in a presynaptic network or originated from a network of gap junction coupled RGCs, we applied the excitatory synaptic blockers NBQX and AP5. Excitatory synaptic blockers eliminated spontaneous activity and spontaneous MI between RGC pairs indicating that spontaneous activity comes from a presynaptic network (Figure 7B; *p* = 0.0147). Given, the temporal characteristics of the spontaneous oscillations in control (Borowska et al., 2011; Haq et al., 2014; Euler and Schubert, 2015) we conclude that the AII-CBC network is the most likely source for spontaneous activity in *rd10* RGCs.

### Influence of distance on shared spontaneous activity

**FIGURE 8:**
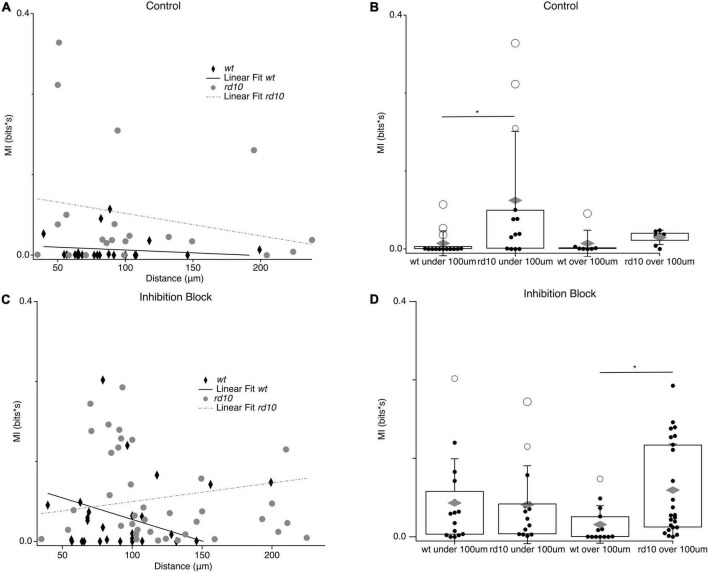
Relationship between MI and inter-pair distance. **(A)** Scatter plots for *wt* (black diamonds) and *rd10* (grey circles) RGC pair spontaneous MI time plotted against inter-pair distance. Solid black line and broken grey line shows the linear fit for *wt* and *rd10*, respectively. **(B)** Binned spontaneous MI under 100 μm and over 100 μm in control conditions for both *wt* and *rd10*. Filled black circles represent data points, open circles represent outliers, whiskers show 1 standard deviation, gray diamonds represent the mean. **(C,D)** Same as **(A,B)** but for in inhibition block (5 μM SR-95531, 50 μM TPMPA, and 5 μM strychnine). *Denotes *p* < 0.05.

The spatial extent of synchronous spontaneous activity over the retina has important implications for population coding and vision restoration efforts. Recent work showed that *rd10* RGC activity showed correlations between pairs up to 400 μm away in an analysis with 200 μm resolution (Ahn et al., 2022), but how coordinated spontaneous activity was modulated by distance at a finer scale was not investigated. To resolve differences in spontaneous MI on a scale closer to the intended resolution of vision restoration technologies, we plotted the MI for pairs as a function of distance (Figure 8). For *wt* pairs, spontaneous MI was low and linear regression did not show a slope significantly different from zero. For *rd10* RGC pairs, spontaneous MI across pairs did show a decreasing trend with distance although the linear regression was not significantly different from zero indicating that activity between RGC pairs is coordinated over long distances in the *rd10* retina. To better quantify the highly heterogeneous MI we divided pairs into groups with inter-pair distances of greater or less than 100 μm (Figure 8B). Spontaneous MI in rd10 was not significantly different between under and over 100 μm pairs, consistent with the none-significant slope of the linear regression. However, spontaneous MI was significantly higher for close *rd10* pairs compared to *wt* (*p* = 0.046, *T*-test, Figure 8B). Again suggesting a weak trend in declining MI with distance for *rd10* pairs that may be obscured by heterogeneity in RGCs responses.

Since inhibition is important for shaping spatial receptive fields (Cook and McReynolds, 1998; Taylor, 1999; Flores-Herr et al., 2001) and in decorrelating spatial input (Franke et al., 2017), we compared how inhibition influenced the spatial extent of spontaneous activity coupling between pairs in *rd10* and *wt*. In *wt* RGC pairs, we observed a selective increase in spontaneous MI for close RGC pairs (*p* = 0.03, *T*-test; Figure 8D) during inhibition block, while pairs over 100 μm showed a smaller increase that did not reach significance. This is consistent with a role for inhibition in actively decorrelating activity between near RGCs with possibly with overlapping receptive fields (Franke et al., 2017), but plays less of a role in preventing the spread of correlated activity over larger distances. It is also interesting to note that with inhibition blocked, the spatial extent of spontaneous MI in *wt* looks similar to that of *rd10* RGC pairs in control conditions, suggesting that inhibition is no longer able to decorrelate activity for near pairs in the *rd10* circuit (Figure 8).

Interestingly, blocking inhibition in *rd10* had the opposite effect on the MI distance relationship than for *wt*. In *rd10*, blocking inhibition strongly increased spontaneous MI between distant pairs (*p* = 0.0007, *T*-Test; Figure 8D), but did not change MI for pairs under 100 μm. The selective increase in MI for distant *rd10* pairs during inhibition block reversed the slope of the relationship between spontaneous MI and distance, with more shared activity for pairs over 100 μm than under. Thus, without the action of inhibition in the *rd10* retina spontaneous MI spreads over much greater distances.

Taken together these experiments reveal several overlapping deficits in spatial processing in the *rd10* network, which ultimately allows for stimulation to drive spontaneous correlated activity across a much greater spatial extent compared to *wt*. First, spontaneous activity has greater shared information between *rd10* RGCs compared to *wt* RGCs. Second, altered circuit function of the *rd10* retina allow for the spread of correlated spontaneous activity over greater retinal area compared to *wt* retina. Finally, inhibition’s role in the retina to decorrelate evoked activity for RGCs with overlapping receptive fields seems to be disrupted in *rd10* retina, however, inhibition does exert an effect to decorrelate aberrant shared information between distant RGCs.

### Receptive field size is not changed in *rd10* retina

**FIGURE 9:**
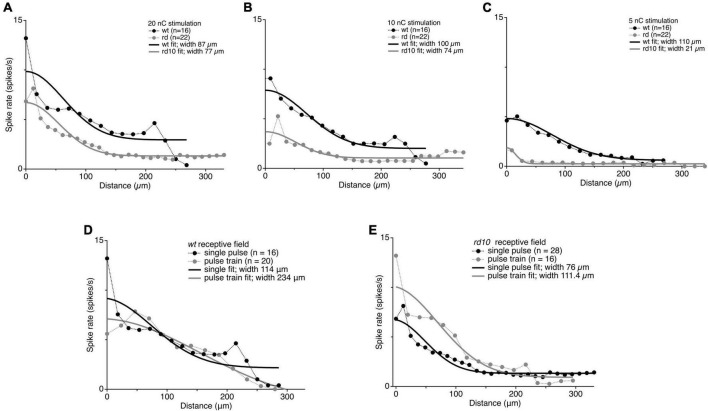
Comparison of receptive field sizes between *wt* and *rd10* at 20 nC **(A)**, 10 nC **(B)**, and 5 nC **(C)**. Black and gray markers show average evoked spike rates for stimulation plotted against distance from stimulating electrode for *wt* and *rd10* RGCs, respectively. Solid lines show the Gaussian fit to the average spike rates for *wt* (black) and *rd10* (gray). Widths were measured as the 1 standard deviation width of the Gaussian fit. Comparison of receptive field size between a single pulse and a 50 Hz pulse train. **(D)** Average evoked spike rates for wt RGCs plotted against distance from stimulating electrode for a single 20 nC pulse (black symbols) and for a 200 ms pulse train at 50 Hz, 20 nC per phase (gray symbols). **(E)** Same as panel **(D)**, but for *rd10* RGCs. Solid lines are the best Gaussian fit to the average data for single pulse (black) and pulse train (gray).

One explanation for the spread of coordinated spontaneous activity in the *rd10* retina is that electrical receptive fields in *rd10* RGCs are generally expanded and integrate activity, whether spontaneous or evoked, over a larger area. To test this hypothesis, we directly measured the electrical receptive fields by presenting electrical stimulation at low (5 nC), medium (10 nC), and strong (20 nC) intensity on stimulation electrodes at varying distance from the target RGC and fit the responses with a gaussian. At 20 and 10 nC stimulation intensities, we found that although rd10 RGCs generated fewer spikes, the width of the receptive field was nearly the same for *wt* and *rd10* retina. Moreover, these widths match well with previously reported receptive field widths for light evoked responses in *wt* RGCs (Kerschensteiner et al., 2008; Figures 9A–C). At the lowest stimulation strength, *wt* electric receptive fields were comparable to the other stimulus intensities, but *rd10* RGCs had a narrower receptive field width. This is likely due to stimulation dropping below threshold for more distant stimuli. Regardless, increases in the spread of synchronized spontaneous activity cannot be explained by a simple increase in spatial summation.

### Lateral spread through the AII/CBC network is low-pass filtered

A simple physiological explanation for the lateral spread of synchronized spontaneous activity over retinal distance is that signals spread laterally through the gap junction coupled network (Ahn et al., 2022); however, this is not consistent with past results showing that gap junction coupling in the inner retinal network is not disrupted, as mentioned above (Euler and Schubert, 2015; Trenholm and Awatramani, 2015, for review), nor is it consistent with our observation that evoked responses do not spread laterally in *rd10* retina. How then can spontaneous activity be synchronized across the retina, while spatial selectivity can be maintained for evoked activity? We hypothesized that the gap junction network may act as a low pass filter, which is capable of spreading slower spontaneous activity between RGCs over longer distances, while maintaining spatial selectivity to fast evoked inputs. To test this, we generated longer lasting electrically evoked activity with a train of stimulation pulses at 50 Hz lasting 200 ms (10 pulses at 20 nC) and measured the RGC receptive field for *rd10* and *wt*. An increase in the width of the electric receptive field was observed for both *wt* and *rd10* RGC’s (114 vs. 234 um; 76 vs. 111.4 um, Figures 9D,E). Consistent with the gap junction network acting as a low pass filter for lateral spread of activity.

## Discussion

Here we show that the ability to encode precise temporal and spatial information from electrical stimulation is preserved in the *rd10* mouse despite increased coordinated firing between RGCs for spontaneous activity. Correlations in spontaneous oscillations have been described before, however, we show for the first time that electrical stimulation synchronizes these oscillations over time and space. The spatial extent of the spontaneous activity is broad compared to normal RGC receptive field sizes. Importantly, we show that despite these large-scale spatial correlations in spontaneous activity, electrically evoked receptive fields were unchanged in *rd10* retina compared to *wt*. We also found that inhibition in the degenerated retina functions in a broadly similarly manner to *wt* retina where it decorrelates spontaneous and evoked activity, even if it cannot fully overcome increase coordinated firing in the *rd10* retina. Together, these data shows that despite extensive circuit dysfunction causing an increase in correlated noise between RGCs, *rd10* retina is still capable of maintaining spatial selectivity, albeit on an elevated noise background.

### Comparison to past results

Much work has described many aspects of spontaneous oscillations in degenerating retina (Margolis and Detwiler, 2011; Euler and Schubert, 2015; Trenholm and Awatramani, 2015) and some recent reports have quantified the spatial extent of correlations in this spontaneous activity between RGCs in degenerating retina (Menzler and Zeck, 2011; Margolis et al., 2014; Ahn et al., 2022). Much less work, however, has characterized the spatial properties of evoked activity in degenerating retina (Lorach et al., 2015b; Stutzki et al., 2016; Ho et al., 2018; Ahn et al., 2022), an important metric when considering vision restoration. A notable case is the recent report by Ahn et al. (2022), which showed that evoked receptive fields in *rd10* retina lost spatial selectivity and would respond to electrodes up to 800 μm away. Other studies have generally found RGC electric receptive fields closer to normal, although these measurements of electric receptive fields for *wt* were also larger than expected, perhaps owing to limitations of the methods used (see below) (Lorach et al., 2015b; Ho et al., 2018). Taken together this leaves much uncertainty about the therapeutic potential for vision restoration strategies that rely on patterning inputs to the remaining retina. Interestingly, we come to a different conclusion. We show that receptive field sizes are unchanged during degeneration in *rd10* retina, and our measurements of both *wt* and *rd10* electric receptive field measurements match closely with expected receptive field size for *wt* light evoked responses (Kerschensteiner et al., 2008).

Given the implications for these findings it is worth exploring possible causes for these disparate findings. First, our experiments and those of Ahn et al. were similar in that biphasic stimulation was delivered on a 30 μm diameter stimulating electrode. The stimulation setup varied more for the other studies, although at a broad level they were similar in that stimulation was delivered through individual or grouped planar stimulating electrodes of a scale suitable for high resolution vision restoration. The range of stimulating charges used in Ahn et al. used 25, 15, and 5 nC, comparable to our, 20, 10, and 5 nC. The stimulation paradigm differed in that we used an anodic first biphasic pulse (Boinagrov et al., 2014; Eickenscheidt and Zeck, 2014) instead of a cathodic first pulse and we delivered stimulating charge over 1 ms per phase instead of 0.5 ms.

Another key difference is in the placement of the stimulating array relative to the retina. Our stimulating electrode array was subretinal, as it was for (Lorach et al., 2015b; Stutzki et al., 2016; Ho et al., 2018), however, Ahn et al. used an epiretinal stimulation paradigm. Comparison of eRF resolution in epiretinal vs. subretinal stimulation configurations has important implications for the field of retinal prosthetics as both strategies have been the subject of significant experimental and pre-clinical interest, and debate over the optimal strategy continues (Goetz and Palanker, 2016; Yue et al., 2016; Damle et al., 2017; Shim et al., 2020). Although epi-retinal approaches have yielded poor spatial control over stimulation due to stimulation of passing axons (Horsager et al., 2010; Ahuja et al., 2013; Weitz et al., 2013), it may be possible to limit this by highly localized, or sinusoidal stimulation (Freeman and Fried, 2011; Weitz et al., 2015; Fan et al., 2019). Moreover, in the case of Ahn et al., it is not clear if this is a contributing factor to the poor eRF resolution in their study given, they attempted to only include indirect network mediated responses.

Another key difference was in the range, resolution, and precision of the measurements. Here we measured spiking responses for a nearly continuous range of stimulation distances between 0 and ∼350 μm from the RGC. In Ahn et al., measurements were made between 200 and 1000 μm with 200 μm resolution, and a 50% of max normalized response was used to define eRF extent, compared to the 1 standard deviation with of a Gaussian fit used on our study and others. Importantly, in all the other studies (Lorach et al., 2015b; Stutzki et al., 2016; Ho et al., 2018; Ahn et al., 2022) cell activity was recorded on an MEA, thus limiting the precision and accuracy of RGC location measurements due to the spacing of electrodes on the MEA, and the inability to determine the location of the RGC from which the activity originates. Our experiments were made using loose-patch recordings from RGCs under IR-DIC microscopy and we could simultaneously visualize both the cell body and stimulation electrode. Therefore, we have measurements of the distance between the stimulating electrode and the RGC cell body with micron resolution. While none of these differences offer a definitive answer to the large discrepancies between the data sets, it is likely that the lack of precise RGC location in the former work could contribute to poor spatial resolution measurements.

Another key difference was in the temporal window used to define evoked responses, and the timing between stimulation pulses. We measured evoked responses as the spikes occurring in the 200 ms after the stimulation pulse, we chose this period because it had the highest peak firing rate often 20–50 ms after stimulation, the peak response typically decayed to baseline within this period and was consistent with evoked responses in *wt* where the lack of spontaneous activity made it obvious where the evoked response ended. One of our key findings in this work is that electrical stimulation synchronizes spontaneous oscillatory spiking, and thus we consistently observed bursts of spontaneous activity occurring within 500 ms of the stimulation pulse. Importantly, we also observed that spontaneous oscillations were stronger and synchronized for several seconds after the stimulation pulse, which led us to wait a minimum of 10 s between stimulation pulses, to ensure a spontaneous oscillation synchronized to the last stimulation pulse did not contaminate our evoked stimulation recording. The looser definition of evoked stimulation (400 ms post stimulus) and higher stimulation repetition rates (1 stimulus per second) used in other studies could conflate evoked responses with regular spontaneous bursts of activity that would occur in the absence of stimulation.

The definition of evoked and spontaneous activity in a spontaneously firing cell maybe somewhat semantic, although it serves to highlight the challenges associated with resolving evoked information carrying visual information, from background activity. The idea that increased spontaneous firing will decrease the signal to noise for restored visual signals has received considerable theoretical and experimental support (Dräger and Hubel, 1978; Sauvé et al., 2001; Stasheff, 2008, 2018; Toychiev et al., 2013; Yee et al., 2014; Barrett, 2015; Ivanova et al., 2016; Telias et al., 2019, 2022; Ho et al., 2020), and to some extent the challenges that exist for experimentally separating evoked and spontaneous spikes will exist for the brain during vision restoration. Our findings show this distinction between evoked and spontaneous activity is important as they have different spatio-temporal properties and are mediated by different circuit mechanisms, and thus may be differentially targeted.

### Implications for the underlying circuitry

Our results clearly indicate that gap junctions in the excitatory network play an important role in both mediating spontaneous oscillatory activity, as well as coordinating activity over distance and through time, consistent with a broad body of literature (Euler and Schubert, 2015; Trenholm and Awatramani, 2015). There is an emerging consensus that spontaneous activity largely arises from oscillations in the gap junction coupled AII-CBC network, but other reports indicate that direct gap junction coupling between RGCs can also contribute (Telias et al., 2019). Our observation that glutamate antagonists block both spontaneous activity and the spread of stimulation evoked synchronization, indicate that the circuit responsible, occurs presynaptic to the RGCs and that ganglion cells themselves do not drive spontaneous activity, at least not without glutamatergic inputs.

Although the biophysical mechanisms that drive this oscillatory network in *rd10* retina have been extensively studied, the implications of these network changes, beyond simply increasing noise, have not been extensively explored. Our findings here not only provide new information about how this disease state will influence vision restoration strategies, but it provides further insight into its underlying physiology. When we observed that electrical stimulation could synchronize spontaneous activity across the retina, we expected that this spatial spread would also extend to evoked responses as well. Paradoxically, we found normal electric receptive fields for *rd10* retina despite the spread of information for spontaneous activity. While it is tempting to suggest that abnormally increased gap junction coupling is responsible for the spread of activity, this interpretation is not supported by past work that found that normal AII-CBC anatomy and circuitry is maintained in the *rd10* retina and oscillations occur when the network loses its inputs (Borowska et al., 2011; Trenholm et al., 2012; Menzler et al., 2014). To explain the discrepancy between evoked electric receptive fields and the spread of spontaneous information, we showed that more sustained activity has a greater lateral spread, consistent with a mechanism where the gap junction coupled AII-CBC network can act as a low pass filter for the lateral spread of spontaneous bursting information in both *wt* and *rd10* retina. This fits well with the widely accepted view of the gap junction network as a low-pass filter in the retina (Curti and O’Brien, 2016). Thus slower spontaneous oscillations propagates laterally through the network to spread information to neighboring RGCs creating spatio-temporal correlations in spontaneous activity, but fast evoked activity does not spread laterally maintaining evoked receptive fields. This may also partially explain why spontaneous information spreads much farther in the presence of inhibition since the oscillations are slowed.

Although this work has focused on the details of spatiotemporal processing in *rd10* retina, our results also provide several new insights into the role and properties of inhibition in degenerated retina, a topic which has received relatively little attention. First, we show that inhibition is capable of performing functions in a broadly similar manner in degenerated *rd10* retina as *wt*. Inhibition is generally thought to decorrelate spatial and temporal information shared between ganglion cells both within and between ganglion cell types (Franke et al., 2017), among other functions, such as gain control (Oesch and Diamond, 2019; Nagy et al., 2021), and we show at a broad level that inhibition decreases correlations and information redundancy between RGCs in both *wt* and *rd10* retina. Our results also point to some interesting differences in the role of inhibition between *wt* and *rd10* retina. In *rd10* retina, inhibition plays a strong role in decreasing long-range correlated activity for more distant RGC pairs but had little effect on the coordinated activity of RGCs located near each other. In contrast, inhibition decorrelated activity in *wt* RGCs near each other, but had little effect on correlations between more distant RGCs. While these observations provide intriguing insight into the function of inhibition during photoreceptor degeneration, these experiments cannot separate normal inhibitory circuit function from the underlying dysfunction in the excitatory circuit, and whether these differences are due to a deficit in inhibition, changes to the underlying excitatory circuit, or a combination of both processes is not certain.

### Implications for vision restoration

The goal of retinal prosthetics is to restore vision in degenerative diseases such as retinitis pigmentosa and age-related macular degeneration by using electrical stimulation to drive activity in spared cells and ultimately recreate spatio-temporal patterns of activity that correspond to useful vision. A central challenge to this effort is that after inputs are lost the remaining circuit undergoes extensive remodeling (Jones et al., 2016), a phenomenon observed throughout the CNS (Turrigiano and Nelson, 2004; Ramocki and Zoghbi, 2008; Martin, 2022). While, changes do occur in the retinal circuit following retinal degeneration, reports of the anatomical, physiological and functional consequences vary widely between particular models, methods, and studies (Lee et al., 2021). Here we chose to focus on the *rd10* mouse model of retinal degeneration as it is well-validated and does not possess any of the developmental confounds of the *rd1* model (Chang et al., 2007; Gargini et al., 2007).

Although mouse retina has notable differences from primate retina, it also shares many similarities, and offers distinct advantages to primate retina. The foremost limitation of primate retina is that suitable models of inherited retinal degeneration are exceedingly rare. In fact, only one such model exists (Moshiri et al., 2019) and it is too rare to meet research needs. Moreover, most primate retinal physiology studies to date are carried out on retina located outside of the fovea, for which mouse retina shares remarkable similarity (Huberman and Niell, 2011), including measures like receptive field size. Finally, the main goal of this paper is to establish how retinal circuitry is changed during degeneration. We identify several key aspects of coding that are changed and are similar, these circuit aspects are well-described in both mouse and primate retina allowing reasonable extension of our findings to retinitis pigmentosa in humans.

It is widely accepted that spontaneous activity will negatively impact vision restoration strategies, such as electronic retinal prosthetics or gene therapy mediated optogenetics, because it will reduce the signal to noise ratio of restored prosthetic visual signals (Dräger and Hubel, 1978; Sauvé et al., 2001; Stasheff, 2008, 2018; Toychiev et al., 2013; Yee et al., 2014; Barrett, 2015; Barrett et al., 2016; Ivanova et al., 2016; Telias et al., 2019, 2022; Ho et al., 2020). Here, we found an additional functional consequence of the disease mediated circuit changes beyond a simple degradation in the signal to noise. Electrical stimulation synchronizes spontaneous activity to a much greater extent in the *rd10* retina compared to *wt* retina, thus reducing the spatial and temporal specificity, as well as the efficiency of retinal coding of visual information. While broader spatio-temporal correlations in spontaneous activity would generally increase information redundancy in the *rd10* retina, the implications for how this will affect the coding of restored visual signals remains unknown.

It is also widely accepted that the limits of spatial resolution in prosthetic vision is determined by the size and spacing of the electrode arrays (Palanker et al., 2005; Wilke et al., 2011). Therefore, there has been significant engineering efforts to decrease pixel size. In roughly a decade pixel spacing has decreased from 575 μm in early retinal prosthetics (Stronks and Dagnelie, 2014), to 100 μm (Palanker et al., 2020) for devices currently in use or in trials. Improvements in resolution are still needed, as one-hundred-micron pixel spacing corresponds to approximately 20/420 visual acuity, still well above the legal definition of blindness (20/200). Recent results provide promise that functional acuity can approach theoretical resolution (Palanker et al., 2020), but a common concern is that circuit rewiring may diminish the functional consequence of further improvements in array resolution, and some past works have supported this concern (Ahn et al., 2022). Our results hold promise for the field of vision restoration as we show that evoked activity can still be restored in degenerated retina with precise spatial control matching that of *wt* retina.

Our results also suggest that there may be strategies to limit the deleterious effects of stimulation on efficient population coding. We found that pathological synchronization of the RGC activity was greatest for stronger stimuli and was reduced at intermediate stimulation intensities. This indicates that we may be able to manipulate the spatio-temporal properties of spontaneous activity correlations by tuning the stimulation intensity, although this would come at the expense of dynamic range (Damle et al., 2021). Moreover, a reduced signal to noise in the face of spontaneous activity also indicates advantages for greater stimulation intensities. Thus, we expect optimal stimulation intensities to be a balance between maximizing signal to noise and dynamic range, while limiting stimulation induced degradation of the spatio-temporal resolution.

## Data availability statement

The raw data supporting the conclusions of this article will be made available by the authors, without undue reservation.

## Ethics statement

This animal study was reviewed and approved by University of California, San Diego, Institutional Animal Care and Use Committee.

## Author contributions

MC conducted the experiments. Both authors conceived the study, analyzed the data, prepared the manuscript, and approved the submitted version.
